# Role of Kinin B_2_ Receptor Signaling in Astrocyte-driven Neuroinflammation

**DOI:** 10.1007/s10571-026-01679-w

**Published:** 2026-01-29

**Authors:** Mariana R. Tavares, Gabriel R. Estrela, Luana Lavezo, Juliene L. S. Silva, Ronaldo C. Araujo, Michael Bader, Frederick Wasinski

**Affiliations:** 1 Department of Neurology and Neurosurgery, Federal University of Sao Paulo, Sao Paulo, 04039-032 Brazil; 2 Department of Biophysics, Federal University of Sao Paulo, Sao Paulo, 04039-032 Brazil; 3 Max-Delbrück Center for Molecular Medicine (MDC), Robert-Rössle-Str. 10, 13125 Berlin, Germany; 4 German Center for Cardiovascular Research (DZHK), Partner Site Berlin, 10117 Berlin, Germany; 5 Institute for Biology, University of Lübeck, Ratzeburger Allee 160, 23562 Lübeck, Germany

**Keywords:** Neuroinflammation, Bradykinin, Kinin B_2_ receptor, Lipopolysaccharide, Astrocytes

## Abstract

The kallikrein–kinin system (KKS) plays a key role in inflammatory responses, but its specific contribution to neuroinflammation remains to be fully elucidated. The bradykinin B_2_ receptor (B_2_R), a principal effector of the KKS, is widely expressed in both neuronal and glial cells in the rodent and human brain. In this study, we investigated the molecular contribution of B_2_R to neuroinflammation using complementary in vitro and in vivo models. Lipopolysaccharide (LPS) stimulation significantly upregulated B_2_R mRNA expression in primary astrocyte cultures and in the cortical tissue of wild-type mice. Pharmacological blockade of B_2_R in astrocytes markedly suppressed the LPS-induced proinflammatory gene expression. In contrast, B_2_R antagonism in vivo resulted in only partial attenuation of the neuroinflammatory response. Together, these findings suggest cell type–specific roles for B_2_R and underscore its key contribution to astrocyte-mediated neuroinflammation.

**Graphical Abstract:**

B_2_R antagonism highlights cell type–specific roles in neuroinflammation. B_2_R pharmacological antagonism suppresses proinflammatory gene expression in astrocytes but shows limited in vivo efficacy, indicating differential functions across brain cell types.

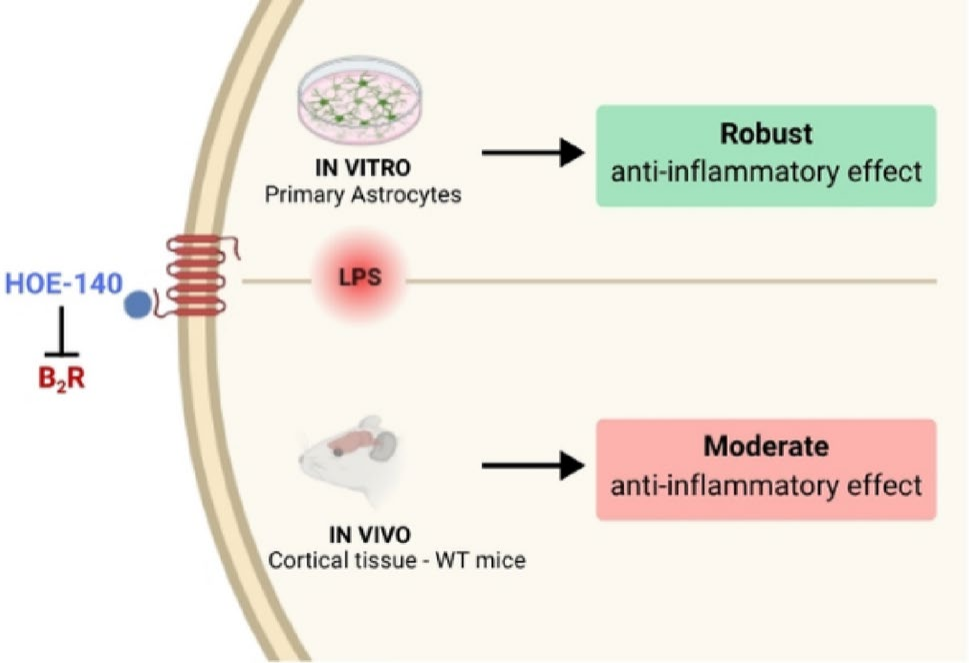

**Supplementary Information:**

The online version contains supplementary material available at 10.1007/s10571-026-01679-w.

## Introduction

Neuroinflammation plays a central role in the pathophysiology of neurodegenerative disorders, including Alzheimer’s disease, Parkinson’s disease, and multiple sclerosis. Within the central nervous system (CNS), this process is mediated by resident glial cells—primarily microglia and astrocytes—which secrete cytokines and chemokines in response to intrinsic or extrinsic stimuli (Skrzypczak-Wiercioch and Sałat 2022). Under physiological conditions, glial cells contribute to key functions such as brain development, synaptic regulation, neuromodulation, and preservation of the blood–brain barrier (BBB). Nonetheless, when exposed to harmful stimuli—including infection, traumatic brain injury, cancer, or stroke—these cells undergo functional changes, adopting a proinflammatory profile characterized by the release of cytokines and reactive oxygen species aimed at limiting tissue damage and restore CNS homeostasis (Skrzypczak-Wiercioch and Sałat 2022).

The kallikrein-kinin system (KKS) is a proteolytic cascade that is well recognized for its activation under inflammatory conditions. It comprises both plasma and tissue kallikreins—enzymes responsible for the hydrolysis of high- and low-molecular-weight kininogens (HMWK and LMWK), resulting in the production of kinins, specifically bradykinin (BK) and kallidin (Lys-BK), respectively. These kinins are subsequently degraded by kininases, such as carboxypeptidases M and N as well as angiotensin-converting enzyme (ACE-I), into either inactive fragments or biologically active carboxy-terminally truncated peptides—des-Arg^9^-BK and des-Arg^10^-kallidin (Dutra 2017). These active metabolites are released at sites of tissue injury, where they function as potent inflammatory mediators by binding to two G protein-coupled receptors: the kinin B_1_ and B_2_ receptors (Sriramula 2020).

The kinin B_1_ receptor (B_1_R) is typically inducible, with its expression significantly upregulated in response to tissue injury or exposure to bacterial endotoxins such as lipopolysaccharide (LPS) (Prado et al. 2002), highlighting its critical role in inflammation. The truncated kinins des-Arg⁹-BK and des-Arg¹⁰-kallidin exhibit high affinity for B_1_R (Dutra 2017). In contrast, the B_2_ receptor (B_2_R) is constitutively expressed under physiological conditions and is preferentially activated by intact BK and kallidin (Regoli and Barabé 1980). Activation of B_1_R and B_2_R triggers various cellular responses, including the release of prostaglandins and nitric oxide (NO), as well as the activation of signaling pathways such as nuclear factor-κB (NF-κB) and mitogen-activated protein kinases (MAPKs), both of which are commonly engaged in cellular stress responses (Dutra 2017).

B_2_R is expressed in multiple regions of both rodent and human brains (Ongali et al. 2003; Mahabeer et al. 2000). However, its functional role within the CNS remains incompletely understood. While some studies report neuroprotective outcomes associated with B_2_R activation (Toricelli et al. 2019; Nunes et al., 2020; Lemos et al. 2010), others suggest that enhanced B_2_R signaling may exacerbate neuroinflammatory responses (Bicca et al. 2015; Dos Santos et al. 2008; Wehn et al. 2024). Given the central role of neuroinflammation in the progression of neurological disorders and the potential involvement of B_2_R in this process, the present study aimed to investigate the molecular contribution of B_2_R to neuroinflammation using both in vitro and in vivo approaches.

## Methods

### Mice

Postnatal day 1 to 2 (P1-P2) C57BL/6 mouse pups were used to isolate cortical cells for astrocytes culture. Eight-week-old male C57BL/6 mice were used for the in vivo assays. Mice were maintained in a 12-h light/dark cycle with ad libitum rodent chow (2.99 kcal/g; 9.4% kcal derived from fat; Nuvilab CR-1, Quimtia, Brazil). To induce acute neuroinflammation, LPS (Sigma-Aldrich, St. Louis, Missouri, USA #L2630) was injected intraperitoneally (5 mg/kg or 10 mg/kg as described). B_2_R antagonist, HOE 140 (400 ug/kg i.p.; Sigma-Aldrich, St. Louis, Missouri, USA #138614-30-9) was injected 24 h, 12 h and 2 h prior to LPS injection (Estrela et al., 2014). This multi-dose regimen was selected because HOE-140 has a short duration of action in vivo and undergoes rapid systemic clearance. Estrela et al., (2014) demonstrated that repeated pre-treatment injections are required to maintain sustained B_2_R antagonism during acute inflammatory challenges. Accordingly, we adopted the same approach to ensure continuous receptor blockade throughout the early LPS-induced inflammatory window. Three hours after LPS injection, the cortical region of the animals was carefully dissected. Mice were randomly assigned to receive intraperitoneal Phosphate Buffered Saline (PBS; Control group, *n* = 4), LPS (LPS group, *n* = 4) and HOE-140 + LPS (HOE-140 + LPS group, *n* = 7) and investigators conducting the experiments were blinded to treatment allocation. The experimental procedures were approved by the Ethics Committee on the Use of Animals of the Universidade Federal de Sao Paulo.

### Primary Mouse Astrocytes Culture

**Fig. 1:**
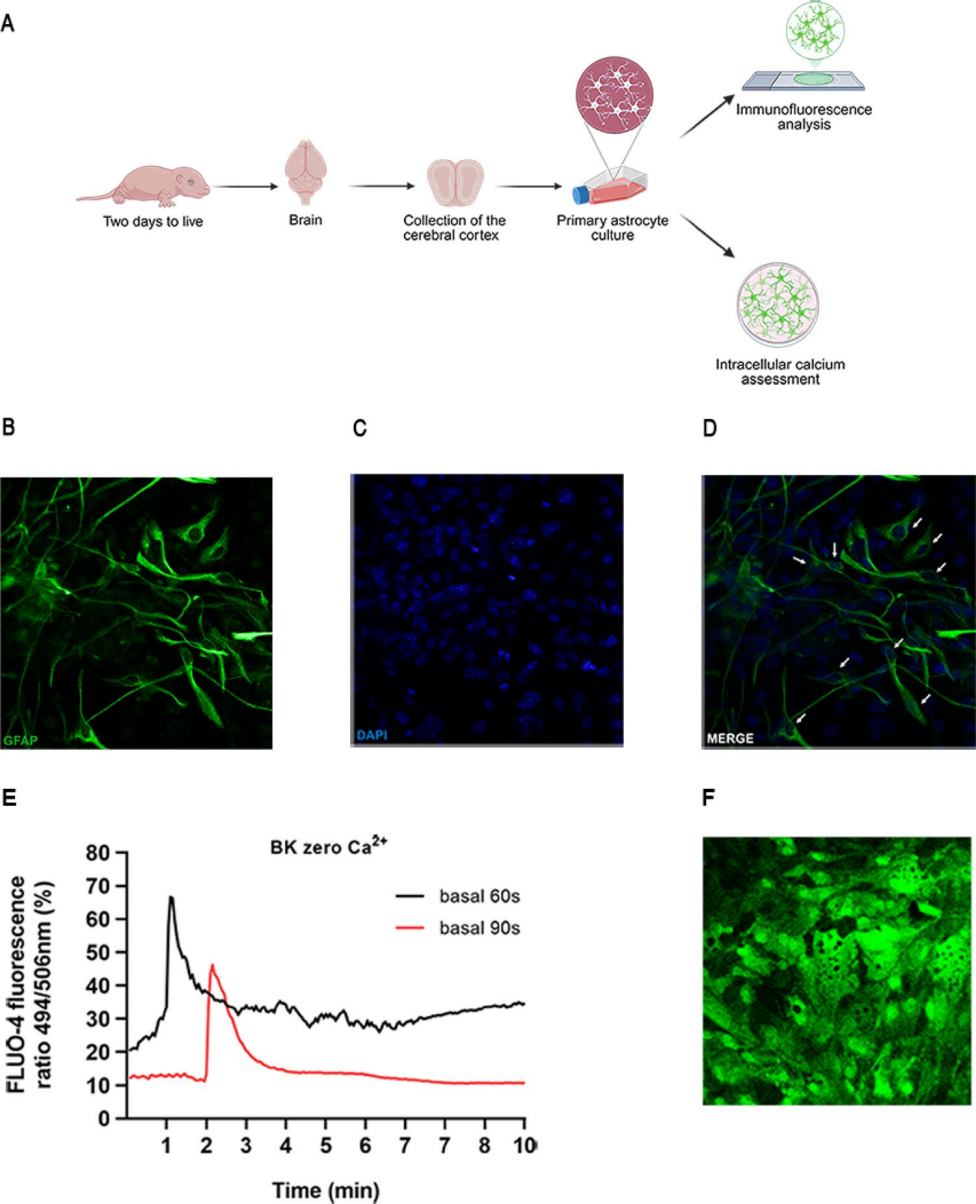
Identification and characterization of primary astrocytes. **A** Schematic representation of the experimental design used to establish primary astrocyte cultures. **B**-**D** Representative images showing GFAP (green), DAPI (blue), and their colocalization (indicated by white arrows) in cultured astrocytes, scale bar: 400 μm. **E** Representative amplitudes obtained from the Fluo-4 fluorescence ratio values (494/506 nm) in the basal state and with BK (1 µg) stimulus after 60 s (black line) and 90 s (red line), illustrating BK-evoked Ca^2+^ signaling. **F** Representative confocal image of astrocyte cells stimulated with BK (1 µg) in the absence of Ca^2 +^ in fluorescence buffer, illustrating calcium release from internal stores, scale bar: 400 μm

To obtain adequate cell density, the cortices from two neonatal mouse pups were carefully isolated and dissected. The tissue was then dissociated and subjected to trypsinization (0.25% trypsin-EDTA, Gibco - Thermo Fisher Scientific, London, United Kingdom #25200-072), as previously described (Schildge et al. 2013). The resulting suspensions were centrifuged at 1200 rpm for 10 min. The pellet was resuspended in 10 mL of DMEM/F-12 Ham medium (Dulbecco’s Modified Eagle Medium, Gibco - Thermo Fisher Scientific, London, United Kingdom #12400024) supplemented with 10% of fetal bovine serum (FBS; Gibco - Thermo Fisher Scientific, London, United Kingdom #12657-029). Cells were then seeded into 75 cm^2^ culture flasks and maintained in an incubator at 37 °C under a 5% CO_2_ atmosphere until reaching semi-confluence (~ 90% of the total surface area). The culture medium was replaced every two days. Once 90% confluence was reached, the cells were considered ready for use in the proposed experiments (Fig. 1A).

To assess the profile of inflammatory and anti-inflammatory gene expression in astrocytes, cells were seeded in 24-well plates and subjected to a starvation protocol in the presence or absence of HOE-140 (10 µM; Sigma-Aldrich, St. Louis, MO, USA; #138614-30-9). After 2 h, the cells were stimulated with LPS (100 ng/mL; Sigma-Aldrich, #L2630) for various time points: 0 h, 6 h, 12 h, and 24 h. The RNA from the cells was subsequently extracted. Biological replicates: Control group, *n* = 5; LPS group, *n* = 6; HOE-140 + LPS group, *n* = 6.

### Identification of Astrocytes in the Cell Culture

Cells were cultured on 13 mm glass coverslips, fixed with 2% paraformaldehyde for 30 min, and subsequently washed with 1X PBS. Coverslips were then immersed in 0.1 M glycine solution (pH 7.4) for 30 min. Non-specific binding sites were blocked using a solution containing 5% FBS, 0.1% Triton X-100, 4% paraformaldehyde, and bovine serum albumin (BSA), followed by overnight incubation with primary antibody: anti-GFAP (Sigma-Aldrich, St. Louis, Missouri, USA #G9269; 1:500) at 4 °C. After incubation, cells were washed and incubated for 1 h at room temperature with secondary antibody Alexa Fluor 488 anti-rabbit (1:250 Thermo Fisher Scientific, London, United Kingdom). Nuclei were counterstained with DAPI (Fluoromount-G, Electron Microscopic Sciences, Hatfield, PA). Photomicrographs were obtained using a confocal microscope (Leica TCS SP8) at 40× magnification (400 μm). Data analyses were performed using LAS EZ software.

### Calcium (Ca^2+^) Measurements After BK Treatment of Astrocytes

Intracellular [Ca^2+^] fluctuations in astrocytes were assessed using the fluorescent dye Fluo-4 AM. Astrocytes were cultured in 25 mm glass coverslips. Cells were pre-incubated with Fluo-4 AM (5 µM; Invitrogen, Carlsbad, California, USA #F14201) and Pluronic F-127 (10%; Sigma-Aldrich, St. Louis, Missouri, USA #P2443) in fluorescence buffer (130 mM NaCl, 5.36 mM KCl, 1 mM MgSO_4_, 1 mM Na_2_HPO_4_, 1.5 mM CaCl_2_, 2.5 mM NaHCO_3_, 1.5 mM albumin, 25 mM glucose, and 20 mM HEPES; pH 7.4) for 30 min. The fluorescence buffer was used either in the presence or absence of Ca^2+^. Fluo-4 AM fluorescence was recorded at 494/506 nm using a real-time fluorescence microscope (Carl Zeiss LSM780) at 40× objective. Baseline fluorescence was acquired for 60 and 90 s, followed by stimulation with BK (1 µg; Sigma-Aldrich, St. Louis, Missouri, USA #B3259). Fluorescence signals were collected for 10 min, with a 3-second interval between images. Data analyses were performed using Zeiss Zen Lite software. Results are expressed as the peak fluorescence intensity of Fluo-4 AM (Zamarioli et al. 2024).

### Quantitative Real-time PCR

Total RNA was extracted from both astrocyte cells and mouse cortex using TRIzol reagent (Invitrogen, Waltham, USA). RNA concentration and purity were evaluated with a NanoDrop One spectrophotometer (Thermo Fisher Scientific, Waltham, USA). To eliminate any residual genomic DNA, samples were treated with RNase-free DNase I (Roche Applied Science, Penzberg, Germany). cDNA synthesis was carried out using 2 µg of total RNA, SuperScript II Reverse Transcriptase (Thermo Fisher Scientific, Waltham, USA), and random hexamer primers p(dN)6 (Roche Applied Science). Quantitative real-time PCR was conducted using the 7500TM Fast Real-Time PCR system (Applied Biosystems, Waltham, USA) and Power SYBR Green (Applied Biosystems) for fluorescence-based detection. Gene expression was analyzed using the 2^−∆∆Ct^ method, normalizing to *Actb* (β-actin) and *Ppia* (cyclophilin A) as reference genes for cortex and *Gapdh* for cells. The primers used are described in Table 1.

**Table 1:** Primer identification and sequences

Target gene	Gene function	Foward primer	Reverse primer
*Actb* (β-actin)	Reference gene	gctccggcatgtgcaaag	catcacaccctggtgccta
*Bdkrb2* (B_2_R)	Bradykinin B_2_ receptor	ggtgctgaggaacaacgaga	cccaacacagcacaaagagc
*Chil3* (YM1)	M2 macrophages marker	cccctggacatggatgactt	agctcctctcaataagggcc
*Gapdh*	Reference gene	gctagccctggacatcgaga	ccccttctttggtgcttttgc
*Il1b* (IL-1β)	Proinflammatory cytokine	gccaccttttgacagtgatg	atgtgctgctgcgagatttg
*Il4r* (IL-4R)	Antiinflammatory marker	cacagtgcacgaaagctgaa	atgggcacaagctgtggtag
*Il6* (IL-6)	Proinflammatory cytokine	tagtccttcctaccccaatttcc	ttggtccttagccactcctcc
*Mrc1* (CD206)	M2 macrophages marker	caaggaaggttggcatttgt	cctttcagtcctttgcaagc
*Nos2* (iNOS)	Nitric oxide production	ctgctggtggtgacaagcacattt	atgtcatgagcaaaggcgcagaac
*Ppia*	Reference gene	cttcttgctggtcttgccattcc	tatctgcactgccaagactgagt
*Tnf* (TNF-α)	Proinflammatory cytokine	gcctcttctcattcctgcttg	ctgatgagagggaggccatt

In addition to the expression of classical pro-inflammatory genes (TNFα, IL-1β, and IL-6), we also assessed the expression of markers associated with alternative macrophage activation and anti-inflammatory responses, such as CD206 (*Mrc1*) and YM1 (*Chil3*), as well as IL-4R, a regulatory receptor involved in modulating glial phenotype.

### Statistics Analysis

Sample size adequacy was determined a priori using G*Power 3.1 software, considering effect size = 0.8, statistical power (1-β = 0.8), and α = 0.05. Data normality was analyzed using the Shapiro-Wilk test and variance homogeneity was assessed prior to applying parametric tests. For datasets following a normal distribution, comparisons among multiple groups were performed using one-way analysis of variance (one-way ANOVA). When significant differences were detected, Tukey’s post hoc analyses were conducted to identify pairwise differences. In cases of non-normal distribution, the non-parametric Kruskal-Wallis test was applied, followed by Dunn’s post hoc analyses. Results are expressed as mean ± standard error of the mean (SEM), and statistical significance was defined as *p* < 0.05. All bar graphs display individual data points overlaid on the bars. All statistical analyses were carried out using GraphPad Prism software, version 8.0.1 (GraphPad Software, San Diego, CA, USA). Group allocation was randomized, and data analysis was performed by an investigator blinded to group identity to minimize subjective bias. Complete statistical information and sample size of each figure are described in Supplementary Table 1.

## Results

1. BK-Induced Intracellular Calcium Signaling in Primary Astrocytes Cultures.

The experimental design used to obtain primary astrocyte cultures is illustrated in Fig. 1A. To characterize the obtained cells, we performed immunofluorescence to assess the expression of glial fibrillary acidic protein (GFAP), a marker of astrocytes, and conducted an assay to evaluate changes in intracellular Ca^2+^ levels, given that bradykinin (BK) induces a transient increase in intracellular Ca^2+^ concentration through activation of B_2_R (17). Figure 1B-D shows GFAP expression (green) in the culture, indicating that the cell population is predominantly composed of astrocytes. Figure 1E demonstrates the effect of BK on astrocytes, showing peaks of FLUO-4 fluorescence beginning at 60 s (black) and 90 s (red), coinciding with BK administration and indicating a BK-induced increase in intracellular [Ca^2+^]. Figure 1F shows a representative image of astrocytes exposed to BK, illustrating increased Fluo-4 fluorescence intensity in nearly all cells following BK stimulation.

2. Pharmacological blockade of B_2_R signaling inhibits LPS-induced inflammatory effects on gene expression in astrocytes.

**Fig. 2:**
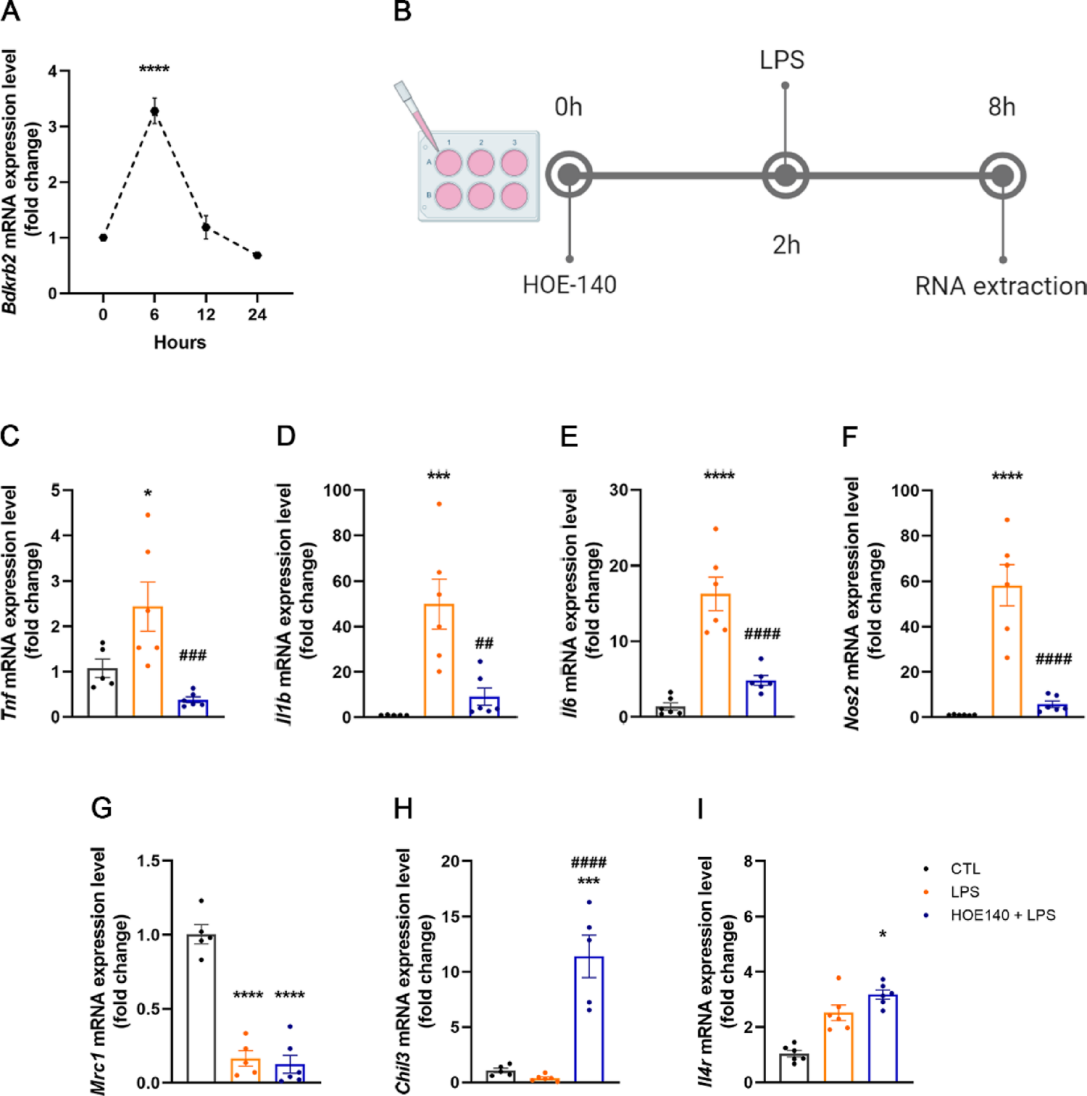
Pharmacological blockade of B_2_R inhibits LPS-induced inflammatory effects on gene expression in astrocytes. **A** Time-course analysis of B_2_R gene expression in astrocytes following treatment with LPS (100 ng/mL) for 6, 12–24 h (*n* = 5–6). **B** Schematic illustration of the experimental design used for evaluate the effects of HOE-140 (10 µM) and LPS (100 ng/mL) induced inflammation in astrocyte culture. **C**-**F** Relative mRNA expression of pro-inflammatory markers (TNF-α, IL-1β, IL-6, iNOS) in astrocytes treated with LPS alone (100 ng/mL) or in combination with HOE-140 (10 µM); (*n* = 6). **G**-**I** Expression of anti-inflammatory markers (CD206, YM1, IL-4R) under the same treatment conditions (*n* = 5–6). Data are expressed as mean ± SEM. * *p* < 0.05; *** *p* < 0.001; **** *p* < 0.0001 vs. control group. ## *p* < 0.01; ### *p* < 0.001; #### *p* < 0.0001 vs. LPS group

To investigate the role of B_2_R in an inflammatory context in astrocytes, we conducted a time-course analysis of LPS treatment to determine the peak of LPS-induced B_2_R gene expression. B_2_R mRNA levels were assessed at 6 h, 12 h, and 24 h following LPS stimulation. We observed that B_2_R expression peaked at 6 h post-LPS treatment (Fig. 2A). Accordingly, all subsequent analyses in astrocytes were performed 6 h after LPS exposure, with or without a 2-hour pretreatment with HOE-140 (Fig. 2B). LPS treatment significantly increased the expression of all analyzed inflammatory markers in astrocytes, including TNF-α (Fig. 2C), IL-1β (Fig. 2D), IL-6 (Fig. 2E) and iNOS (Fig. 2F). This effect was completely abolished by blocking B_2_R signaling with HOE-140 (Figs. 2C-F). Moreover, the absence of B_2_R signaling in astrocytes resulted in increased expression of the anti-inflammatory gene YM1 (Fig. 2H). Therefore, we conclude that blocking B_2_R signaling in astrocytes was effective in reducing the intensity of LPS-induced inflammation by modulating the expression of pro-inflammatory markers and, to a lesser extent, promoting the expression of anti-inflammatory genes (Fig. 2H and I).

3. Pharmacological blockade of B_2_R signaling partially attenuates LPS-induced inflammatory gene expression in mouse cortical tissue.

**Fig. 3:**
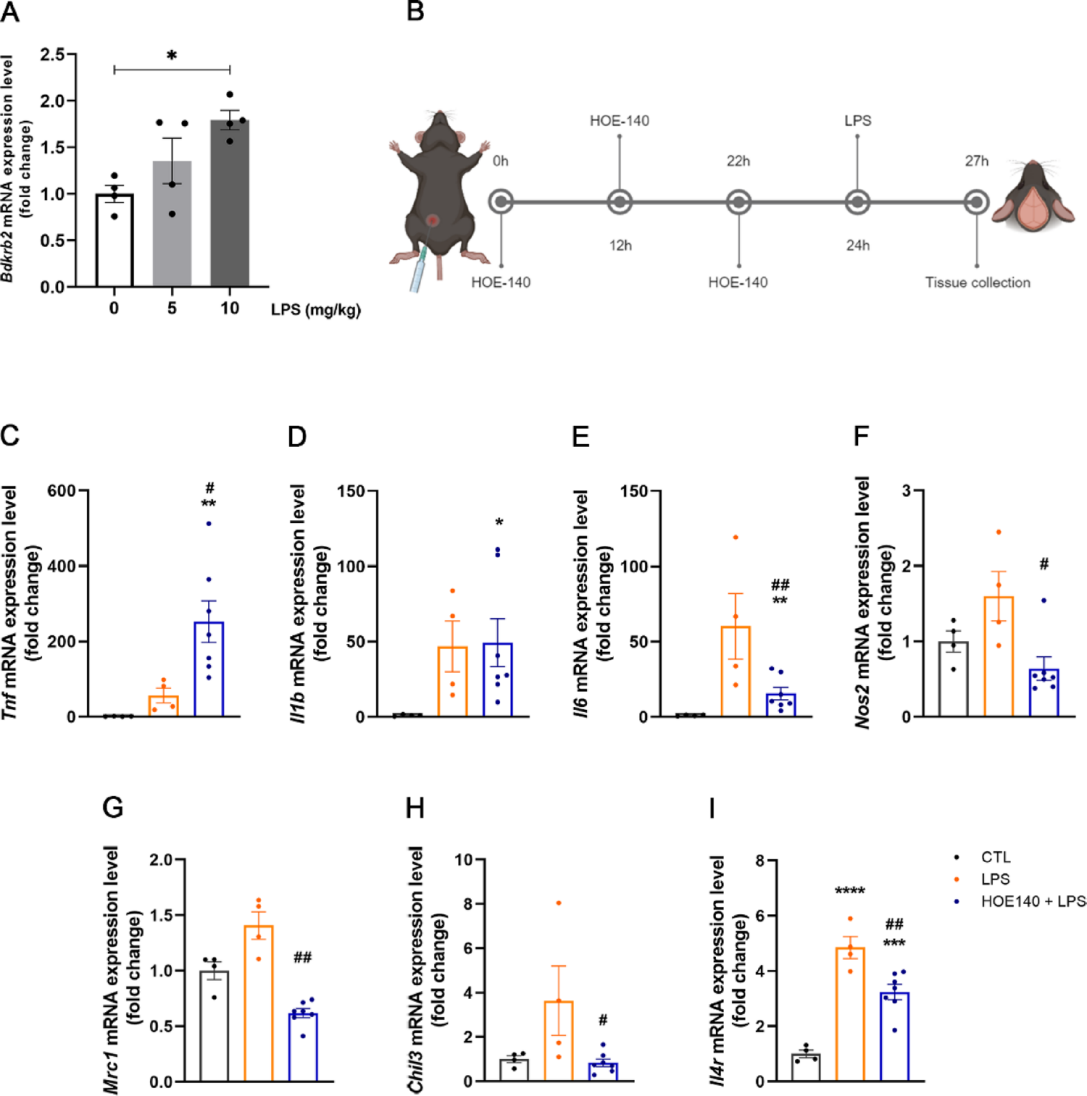
Pharmacological blockade of B_2_R partially attenuates LPS-induced inflammatory gene expression in mouse cortical tissue. **A** B_2_R gene expression in the cortical tissue of mice intraperitoneally treated with two doses of LPS (5 and 10 mg/kg), compared to the control group (*n* = 4/group). **B** Schematic illustration of the experimental design used for treating animals with HOE-140 (400 ug/kg i.p.) and LPS (10 mg/kg i.p.). **C**-**F** Relative expression of pro-inflammatory markers (TNF-α, IL-1β, IL-6, iNOS) in the cortex of mice treated with LPS alone (10 mg/kg i.p.) or in combination with HOE-140 (400 ug/kg i.p.); (*n* = 4–7). **G**-**I** Expression of anti-inflammatory markers (CD206, YM1, IL-4R) in the same treatment groups (*n* = 4–7). Data are presented as mean ± SEM. * *p* < 0.05; ** *p* < 0,01; *** *p* < 0.001; **** *p* < 0.0001 vs. control group. # *p* < 0.05; ## *p* < 0.01 vs. LPS group

To evaluate the effects of pharmacological inhibition of B_2_R signaling on neuroinflammation in vivo, adult mice were treated with either a moderate (5 mg/kg ip) or a high (10 mg/kg ip) dose of LPS. Although the moderate LPS dose has been reported to induce neuroinflammation in adult mice (Hoogland et al. 2015), we selected the higher dose for the subsequent experiments because only the 10 mg/kg treatment consistently induced a significant increase in B_2_R mRNA levels in the cortex of young adult mice (Fig. 3A). This higher dose is used in the literature to model acute systemic inflammation capable of triggering central nervous system inflammatory cascades, including cytokine upregulation, microglial activation, and sickness behavior (Hoogland et al. 2015; Bhaskar et al. 2010). Besides, data suggest that LPS-induced neuroinflammation in rodents can require mg-range doses to elicit reliable cytokine induction and CNS inflammatory signaling (Catorce and Gevorkian 2016; Silva et al., 2024; Fu et al. 2014). Therefore, 10 mg/kg LPS provides a physiologically relevant and reliably reproducible model of acute neuroinflammation, which is necessary to investigate the contribution of B_2_R signaling in this context.

Figure 3B illustrates the experimental design employed for the treatment of mice. HOE-140 was administered intraperitoneally three times prior to LPS injection, with 12- and 10-hour intervals between each administration. In contrast to the in vitro experiments, cortical tissue in vivo displays a rapid inflammatory response, with robust induction of cytokines and inflammatory mediators occurring within 1–3 h after systemic LPS administration (Qin et al., 2007; Lee et al. 2008). For this reason, cortical tissue was analyzed 3 h after LPS treatment (Fig. 3B).

Unlike the pronounced effect of LPS in astrocytes, its induction of inflammatory gene expression in the cortex was relatively modest (Figs. 3C–F). TNF-α expression was unexpectedly elevated in the cortices of animals pretreated with HOE-140 compared to both the control and LPS groups (Fig. 3C). Besides, HOE-140 had no significant effect on IL-1β gene expression relative to the LPS group (Fig. 3D). On the other hand, HOE-140 effectively reduced the expression of the inflammatory markers IL-6 and iNOS, which were upregulated by LPS treatment in the cortex (Fig. 3E and F). Additionally, HOE-140 pretreatment decreased cortical expression of the anti-inflammatory genes CD206, YM1, and IL-4R compared to the LPS group (Figs. 3G–I). Therefore, we conclude that the blockade of B2R signaling in vivo is less effective in suppressing the LPS-induced exacerbated inflammatory gene expression profile than its direct effect on astrocytes.

## Discussion

In this study, we investigated the role of the kinin B_2_ receptor (B_2_R) in modulating the inflammatory response triggered by lipopolysaccharide (LPS) using both in vitro and in vivo models. Our findings indicate that B_2_R signaling plays a key role in promoting pro-inflammatory gene expression in primary astrocyte cultures and, to a lesser extent, in the cortical tissue of adult mice. Furthermore, we demonstrate that pharmacological inhibition of B_2_R with HOE-140 effectively suppresses LPS-induced inflammation in astrocytes, while eliciting partial anti-inflammatory effects in the cortex.

This pro-inflammatory profile is consistent with previously reported mechanisms. For instance, Levant et al. (2006) reported that bradykinin stimulation of B_2_R in glial cells enhances prostaglandin E2 (PGE2) synthesis, especially under LPS-induced inflammatory conditions. In line with these findings, our model showed that activation of the LPS-B_2_R axis upregulated inflammatory markers in astrocytes, and this response was significantly attenuated by B_2_R antagonism. Collectively, these data support the hypothesis that B_2_R functions as an amplifier of glial inflammatory responses and may represent a therapeutic target in conditions of exacerbated neuroinflammation.

Beyond its classical vascular role, B_2_R signaling may also influence neuronal function through astrocyte-derived mediators. Liu et al. (2009) demonstrated that B_2_R activation in astrocytes promotes glutamate release via reactive oxygen species (ROS) generation and activation of volume-sensitive outwardly rectifying (VSOR) Cl⁻ channels. Glutamate release may exacerbate neuronal excitability and contribute to excitotoxicity under sustained inflammatory conditions. Our findings complement this work by showing that B_2_R activation also promotes cytokine expression in astrocytes, while its inhibition mitigates inflammatory responses. These complementary roles suggest that B_2_R acts at the interface between neuroinflammation and synaptic dysfunction. Considering the pivotal role of glutamate in excitatory transmission and neuronal injury, targeting specific B_2_R signaling in the astrocytes could offer a dual benefit: reducing inflammatory signaling and preserving neuronal integrity. Thus, selective B_2_R inhibition emerges as a promising therapeutic strategy to limit glial-driven damage in neuroinflammatory contexts.

Extending beyond acute models, B_2_R has also been implicated in chronic neuroinflammatory diseases. Consistent with our results, Dutra et al. (2013) showed that both pharmacological blockade and genetic deletion of B_2_R reduce glial activation and pro-inflammatory cytokine expression in a murine model of multiple sclerosis (EAE). Although our study utilized an acute LPS-driven paradigm, both lines of evidence underscore the central role of B_2_R signaling in shaping glial immune responses under diverse pathological conditions. Furthermore, the involvement of B_2_R in astrocyte activation appears to be conserved across species and immune challenges. For example, Kim et al. (2010) demonstrated that bradykinin triggers the expression of inflammatory cytokines in human astrocytes (1321N1 cell line) following zymosan stimulation, via MAPK and NF-κB activation. These findings align with our observations in murine primary astrocytes, in which HOE-140 reduced LPS-induced expression of TNF-α, IL-1β, IL-6 and iNOS. Such convergence strengthens the evidence for B_2_R as a mediator of glial immune responses to various pro-inflammatory stimuli.

We also observed that inhibition of B_2_R signaling, both in vitro and in vivo, resulted in decreased IL-6 and iNOS mRNA expression, consistent with previous studies showing that bradykinin stimulates IL-6 secretion and gene expression in astrocytes via NF-κB activation (Schwaninger et al. 1999), as well as the established mechanism by which bradykinin, through B_2_R activation, stimulates iNOS expression to promote nitric oxide production and subsequent vasodilation (Dutra 2017). These findings further support the involvement of B_2_R in NF-κB–mediated cytokine production and position this receptor as a viable target for controlling astrocyte-driven inflammation in the CNS.

The anti-inflammatory effect of HOE-140 observed in the cortex of adult mice was less pronounced compared to that seen in isolated astrocytes. In contrast to the findings in vitro, HOE-140 treatment in vivo failed to reverse the LPS-induced upregulation of TNF-α and IL-1β, key proinflammatory cytokines involved in the acute phase of inflammation (Taishi et al. 2008)—corresponding to the specific time point at which cortical tissue was analyzed. Moreover, disruption of B_2_R signaling reduced the expression of anti-inflammatory markers in vivo. These data may suggest a functional role for B_2_R signaling in neurons, consistent with previously reported neuroprotective effects observed in neurons isolated from young mice (Toricelli et al. 2019). The discrepancy between the in vitro and in vivo findings may be explained by the cellular heterogeneity of the cortical environment, which comprises a variety of neuronal and glial cell types, each potentially responding differently to B_2_R signaling. An important factor contributing to the discrepancy is that the cortex is a heterogeneous tissue composed of neurons, astrocytes, microglia, and endothelial cells, each expressing B_2_R and responding to LPS with distinct signaling dynamics. In purified astrocyte cultures, B_2_R antagonism directly reduces pro-inflammatory pathways; however, in vivo, neuronal B_2_R activation has been shown to attenuate excitotoxicity and limit inflammatory amplification (Sarit et al. 2012). Blocking this neuroprotective component may unmask or even potentiate microglial TNF-α production, explaining why TNF-α increases after HOE-140 treatment despite the attenuating effects observed in vitro. Likewise, IL-1β expression may be less sensitive to B2R blockade in a mixed-cell environment, where multiple regulatory circuits converge. These results reinforce the idea that the contribution of the kallikrein–kinin system (KKS) to neuroinflammation is highly context-dependent, varying according to cell type, developmental stage, and the specific cellular milieu involved (Toricelli et al. 2019).

Importantly, several studies demonstrate that B_2_R activation can exert neuroprotective effects depending on stimulus intensity and cellular context. In models of ischemia and inflammatory injury, B_2_R signaling has been shown to reduce neuronal loss, modulate oxidative stress, and promote tissue recovery (Ji et al. 2017, 2019). These findings support the notion that B_2_R participates in homeostatic mechanisms that balance inflammatory signaling and neuronal survival, offering a plausible explanation for the region- and cell type–dependent transcriptional patterns observed in our study.

In addition, we investigated the role of B_2_R signaling in an acute neuroinflammatory state in young adult mice. While our study focused on this specific context, previous research has demonstrated a neuroprotective role for B_2_R in aging and neurodegenerative diseases, such as Alzheimer’s disease (Caetano et al., 2015; Nunes et al., 2020; Ji et al. 2019), including improvements in certain cognitive parameters—which were not assessed in our model. Nonetheless, the effects of B_2_R signaling in neurological disorders remain controversial, particularly concerning its function in neurons and glial cells, as thoroughly reviewed by Nokkari et al. (2018). Moreover, B_2_R signaling can engage multiple downstream pathways, such as ERK1/2–apelin/APJ or ERK1/2–NF-κB, which may play distinct roles in neuroprotection and neuroinflammation, respectively (Ji et al. 2019). Although the involvement of the kallikrein–kinin system (KKS) in neuroinflammation is well established, the specific contribution of each component requires further investigation to clarify the conflicting findings reported in the current literature. The use of available genetic models offers a promising strategy to dissect these complexities across different neuroinflammatory contexts. Alternatively, co-culture systems comprising neurons and glial cells offer a controlled in vitro environment to further investigate the role of the KKS in neuroinflammatory processes.

It is also important to note that no commercially available antibodies reliably detect B_1_R or B_2_R in mouse tissue, a limitation widely recognized in the kinin receptor field. This constraint restricts the feasibility of protein-level assays such as Western blot or immunohistochemistry, making qPCR analysis and pharmacological manipulation with a selective B_2_R antagonist (HOE-140) the most robust and reproducible approaches currently available to assess receptor involvement.

Taken together, our findings highlight the pivotal role of B_2_R in astrocyte-driven neuroinflammation, reinforcing evidence from both acute and chronic models of CNS injury. Limiting the persistent activation of glial cells is crucial to prevent neuronal damage and, consequently, neurodegeneration (Qin et al., 2007). Our data identify B_2_R as a potential therapeutic target, specifically in astrocytes, to mitigate this pathological process. Future studies are warranted to determine whether targeting B_2_R can attenuate long-term neuroinflammatory damage and preserve CNS homeostasis in disease models.

## Supplementary Information

Below is the link to the electronic supplementary material.

Supplementary Material 1

## Data Availability

The datasets generated during and/or analysed during the current study are available in the UNIFESP repository, https://repositorio.unifesp.br/.
