# Supplementary material for: Role of Kinin B2 Receptor Signaling in Astrocyte-driven Neuroinflammation
Source: Cell Mol Neurobiol. 2026 Jan 29;46:38. doi: 10.1007/s10571-026-01679-w (PMC12905013; doi:10.1007/s10571-026-01679-w)
Supplement: Supplementary file 1 — Supplementary Material 1 [file 10571_2026_1679_MOESM1_ESM.docx]

**Supplemental Table 1. Summary of statistical analyses.**

| Figure | Sample size | Statistical test | P values |
| --- | --- | --- | --- |
| 2a | 0 (n = 5)  6 (n = 6)  12 (n = 5)  24 (n = 6) | One-way ANOVA and Tukey’s multiple comparisons test | F_(3, 18)_ = 2.076, *P* < 0.1392  Multiple comparisons:  0 vs. 6: P < 0.001  0 vs. 12: P = 0.8724  0 vs. 24: P = 0.5226  6 vs. 12: P < 0.001  6 vs. 24: P < 0.001  12 vs. 24: P = 0.1671 |
| 2c | Control (n = 5)  LPS (n = 6)  HOE140 + LPS (n = 6) | One-way ANOVA and Tukey’s multiple comparisons test | F_(2, 14)_ = 4.398, *P* = 0.0330  Multiple comparisons:  Control vs. LPS: P = 0.0440  Control vs. HOE140 + LPS: P = 0.3817  LPS vs. HOE140 + LPS: P = 0.0022 |
| 2d | Control (n = 5)  LPS (n = 6)  HOE140 + LPS (n = 6) | Kruskal-Wallis test and Dunn’s multiple comparisons test | *P* < 0.0001  Multiple comparisons:  Control vs. LPS: P = 0.0006  Control vs. HOE140 + LPS: P = 0.1911  LPS vs. HOE140 + LPS: P = 0.1554 |
| 2e | Control (n = 5)  LPS (n = 6)  HOE140 + LPS (n = 6) | One-way ANOVA and Tukey’s multiple comparisons test | F_(2, 15)_ = 7.723, *P* = 0.0049  Multiple comparisons:  Control vs. LPS: P < 0.0001  Control vs. HOE140 + LPS: P = 0.2074  LPS vs. HOE140 + LPS: P < 0.0001 |
| 2f | Control (n = 5)  LPS (n = 6)  HOE140 + LPS (n = 6) | One-way ANOVA and Tukey’s multiple comparisons test | F_(2, 15)_ = 7.995, *P* = 0.0043  Multiple comparisons:  Control vs. LPS: P < 0.0001  Control vs. HOE140 + LPS: P = 0.8083  LPS vs. HOE140 + LPS: P < 0.0001 |
| 2g | Control (n = 5)  LPS (n = 6)  HOE140 + LPS (n = 6) | One-way ANOVA and Tukey’s multiple comparisons test | F_(2, 13)_ = 0.02275, *P* = 0.9775  Multiple comparisons:  Control vs. LPS: P < 0.0001  Control vs. HOE140 + LPS: P < 0.0001  LPS vs. HOE140 + LPS: P = 0.8880 |
| 2h | Control (n = 5)  LPS (n = 6)  HOE140 + LPS (n = 6) | One-way ANOVA and Tukey’s multiple comparisons test | F_(2, 13)_ = 6.739, *P* = 0.0098  Multiple comparisons:  Control vs. LPS: P = 0.8802  Control vs. HOE140 + LPS: P < 0.0001  LPS vs. HOE140 + LPS: P < 0.0001 |
| 2i | Control (n = 5)  LPS (n = 6)  HOE140 + LPS (n = 6) | One-way ANOVA and Tukey’s multiple comparisons test | F_(2, 15)_ = 0.7077, *P* = 0.5085  Multiple comparisons:  Control vs. LPS: P = 0.0003  Control vs. HOE140 + LPS: P < 0.0001  LPS vs. HOE140 + LPS: P = 0.0816 |
| 3a | 0 (n = 5)  5 (n = 6)  10 (n = 6) | One-way ANOVA and Tukey’s multiple comparisons test | F_(2, 9)_ = 5.531, *P* = 0.0271  Multiple comparisons:  0 vs. 5: P = 0.3206  0 vs. 10 + LPS: P = 0.0180  5 vs.10: P = 0.1896 |
| 3c | Control (n = 4)  LPS (n = 4)  HOE140 + LPS (n = 7) | One-way ANOVA and Tukey’s multiple comparisons test | F_(2, 12)_ = 3.840, *P* = 0.0514  Multiple comparisons:  Control vs. LPS: P = 0.7391  Control vs. HOE140 + LPS: P = 0.0063  LPS vs. HOE140 + LPS: P = 0.0287 |
| 3d | Control (n = 4)  LPS (n = 4)  HOE140 + LPS (n = 7) | Kruskal-Wallis test and Dunn’s multiple comparisons test | *P* = 0.0070  Multiple comparisons:  Control vs. LPS: P = 0.0656  Control vs. HOE140 + LPS: P = 0.0192  LPS vs. HOE140 + LPS: P > 0.9999 |
| 3e | Control (n = 4)  LPS (n = 4)  HOE140 + LPS (n = 7) | One-way ANOVA and Tukey’s multiple comparisons test | F_(2, 12)_ = 6.116, *P* = 0.0147  Multiple comparisons:  Control vs. LPS: P = 0.0093  Control vs. HOE140 + LPS: P = 0.5915  LPS vs. HOE140 + LPS: P = 0.0243 |
| 3f | Control (n = 4)  LPS (n = 4)  HOE140 + LPS (n = 7) | Kruskal-Wallis test and Dunn’s multiple comparisons test | *P* = 0.0176  Multiple comparisons:  Control vs. LPS: P > 0.9999  Control vs. HOE140 + LPS: P = 0.2930  LPS vs. HOE140 + LPS: P = 0.0325 |
| 3g | Control (n = 4)  LPS (n = 4)  HOE140 + LPS (n = 7) | Kruskal-Wallis test and Dunn’s multiple comparisons test | *P* < 0.0001  Multiple comparisons:  Control vs. LPS: P = 0.6177  Control vs. HOE140 + LPS: P = 0.1492  LPS vs. HOE140 + LPS: P = 0.0021 |
| 3h | Control (n = 4)  LPS (n = 4)  HOE140 + LPS (n = 7) | One-way ANOVA and Tukey’s multiple comparisons test | F_(2, 12)_ = 4.632, *P* = 0.0323  Multiple comparisons:  Control vs. LPS: P = 0.0916  Control vs. HOE140 + LPS: P = 0.9855  LPS vs. HOE140 + LPS: P = 0.0412 |
| 3i | Control (n = 4)  LPS (n = 4)  HOE140 + LPS (n = 7) | One-way ANOVA and Tukey’s multiple comparisons test | F_(2, 12)_ = 0.7801, *P* = 0.4803  Multiple comparisons:  Control vs. LPS: P < 0.0001  Control vs. HOE140 + LPS: P = 0.0005  LPS vs. HOE140 + LPS: P = 0.0061 |
